# Allelopathic pathways and impacts of Chenopodium species via leachates, decaying residues, and essential oils

**DOI:** 10.1371/journal.pone.0321782

**Published:** 2025-04-29

**Authors:** Abeer Al-Andal, Asmaa M. Radwan, AbdElRaheim M. Donia, Mohamed A. Balah

**Affiliations:** 1 Department of Biology, College of Science, King Khalid University, Abha, Saudi Arabia; 2 Botany and Microbiology Department, Faculty of Science, Girls Branch, Al-Azhar University, Cairo, Egypt; 3 Medicinal and Aromatic Plants Department, Desert Research Center, Cairo, Egypt; 4 Plants Protection Department, Desert Research Center, Cairo, Egypt; Canakkale Onsekiz Mart University, TÜRKIYE

## Abstract

The potentials of Chenopodium species are important for both the environment and agricultural production. To comprehend their effects, their allelopathic pathways were investigated. The impacts of *C. album*, *C. murale*, and *C. ambrosioides* weeds were compared via leachates of water extracts, decaying residues, and volatilization and measured biologically and chemically on plant traits and soil characteristics. The allelopathic effect of water extracts from the aboveground parts was more potent than that from the subterranean parts, significantly influenced by the plant parts and concentrations. The allelochemicals determined by LC-ESI-MS were coumaric and ferulic acid, with concentrations of 4.74 and 5.72 μg ml^-1^ for *C. album*, 7.07 and 5.71 μg ml^-1^ for *C. murale*, and 8.88 and 4.82 μg ml^-1^ for *C. ambrosioides*, respectively. The allelopathic effect of incorporated residues into soil was affected significantly by plant types, concentration, and their interactions. The essential oils of shoot parts showed the strongest suppression in *B. rigidus* and *C. arvensis* germination and growth. The principal components of essential oils determined by GC-MS were ascaridole (*C. ambrosioides*), carvacrol (*C. murale*), and carvacrol (*C. album*) with concentrations of 8.87%, 10.64%, and 8.43%, respectively. Notably, *C. ambrosioides* and *C. murale* demonstrated the strongest inhibitory effects, followed by *C. album* suggested to be used as herbicide treatments in the future. Chenopodium species exert significant inhibitory effects by releasing allelochemicals against several tested species. Therefore, allelopathy appears to be responsible for the nearby plant structure through the action of their bioactive metabolites.

## Introduction

Chenopodium is a genus of 25 species recognized as weeds in different parts of the world [1]. It belongs to the family Amaranthaceae, syn. Chenopodiaceae [2], includes about 102 genera and 1400 annual herbaceous species with a pungent smell distributed worldwide, especially in the moderate and subtropical zone [3,4]. Chenopodium, L. (family Chenopodiaceae) is a genus of annual to perennial herbaceous plants [5,6]. The Chenopodium genus is not a well-understood complex and many species are highly polymorphic in habit, height, branching, and leaf size [7]. They mostly grow as weeds throughout the world [8,9] with a few exceptions, while, the majority of them are annual weeds [10]. Whereas, some of these weeds are resistant to one or more classes of herbicides [11]. *C. album* and *C. murale* have unique biological features, including high reproductive capacity, seed dormancy, high persistence in the soil seed bank, and the ability to germinate and grow under a wide range of environmental conditions and abiotic stress tolerance, which help these species to infest diverse cropping systems [12].

*Chenopodium album* L., (common lambsquarters) is an annual weed species in more than 40 crop species in 47 countries [13]. It is a troublesome weed in spring-planted crops throughout the world [14,15]. It has a successful character as weed including its ability to germinate under different environmental conditions [16,17]. *C. album* extract decreased soybean, wheat and corn growth [18]. Competitive and, allelopathic effects of *C. album* on tomatoes were successfully segregated by **Quasem & Hill** [19]. The presence of *C. album* residual material in soil caused the growth reduction of wheat [20] and various other crop species [21]. *C. album* is more problematic than other species of the genus, and infests more crops, as well as acts as an alternate host of several crop pests and pathogens [22]. The allelopathic property of *C. album* is presented as one of the damage-full parameters, which is caused by the allelopathic effects of different plant parts [23]. The essential oil of *C. album* had the greatest potential to be considered as an antibacterial agent against MDR bacterial strains. This potential was due to different biological and bioactive compounds like phytol, linalool, α-terpineol and linolenic acid in the plant [24].

*Chenopodium murale* L. is one of the most problematic weeds causing damage across a range of climates. It has rare biological characteristics, ecological acclimatization, being a good competitor, and allelochemical abilities [25]. It is a widely adapted and opportunistic colonizer of disturbed areas that has spread globally via human vectors and the long-term association of humans with agriculture [26]. *C. murale* negatively impacts cropping systems due to its effects on the development and growth of several crop plants, decreasing the ability of biological nitrogen fixation [27]. *C. murale* is particularly damaging from an ecological perspective because of its detrimental effects on the nitrogen fixation process and the growth of beneficial bacteria in the plant’s rhizosphere [28]. *C. murale* is also a fast-growing annual [29] that infests more than 25 crops in different parts of the world [30]. *C. murale* aqueous extracts reduced seed germination, seedling establishment, and plant growth of *Melilotus indicus, Trifolium alexandrinum, Triticum pyramidal, Lycopersicon esculentum,* and *Cucumis sativus.* Similar effects were found for pigment, carbohydrate, and protein contents. In general, the inhibition percentage was a function of extract concentration and plant tissue type [31].

*Chenopodium ambrosioides* L. is an invasive plant native to the Neotropics that has seriously threatened ecological security [32]. The release of allelochemicals by either pathway results in them entering the soil and affecting the growth of surrounding plants [33] and the germination of some cultivated plants was reported [34]. The extract of *C. ambrosioides* was highly effective against engorged females of cattle ticks [35]. Essential oils of *C. ambrosioides* were effective against the phytopathogens *Fusarium oxysporum* and *Colletotrichum gloesporioides* [36]. The essential oil obtained from this species has antihelmintic and antifungal activities [37]. oils with more than 60–70% ascaridole-rich fractions are suitable for commercial exploitation [38].

To preserve agriculture productivity from these weeds, a comprehensive understanding of Chenopodium species allelopathy is needed to manipulate their phytotoxic potentials in crop species. This involves understanding the behavior and pathways of these allelochemicals into the soil environment and addressing their putative concentrations against certain tested species and soil properties. So, the study aims to investigate the allelopathic effects of three Chenopodium species, including the invasive *C. ambrosioides* and the non-invasive *C. album* and *C. murale*, on crop species. We hypothesize that *C. ambrosioides*, as an invasive species, exhibits stronger allelopathic potential than the non-invasive species. These allelopathic effects are expected to negatively influence plant growth and soil properties, posing a significant challenge for agriculture. The study will explore the allelopathic activity of the three species through different pathways, including water-soluble leachates, decaying plant residues, and essential oils. Furthermore, we will identify and compare the concentrations of key allelochemicals among the species. The research will also examine how these allelopathic activities alter soil properties, with the goal of assessing their potential impact on agricultural productivity.

## Materials and methods

### 1. Plant materials

All the steps of experimentation on *Chenopodium album*, *Chenopodium murale*, and *Chenopodium ambrosioides* weeds, including the collection of plant material, were in compliance with relevant institutional, national, and international guidelines. The studies were conducted in accordance with local legislation and with permissions from the Desert Research Center and they complied with the IUCN Policy Statement. The test species were collected during their flowering stages in 2022–2023 from the South Sinai Governorate and subsequently brought to the laboratory of the Plant Protection Department at the Desert Research Center in Cairo, Egypt. A plant taxonomist Dr. Emad Abdel-Kader Desert Research Center, confirmed the identification of the specimens [39]. These weeds were divided into vegetative and subterranean sections, air-dried at room temperature, and stored in glass jars. Wheat (*Triticum aestivum*) Giza-193 cultivar and faba bean (*Vicia faba*) Marryout 2 cultivar were provided by the Agriculture Research Center and Desert Research Center respectively. *Convolvulus arvensis*, also known as bindweed, and *Bromus rigidus* Roth, also known as brome, were gathered from the Marriott Research Station at the Desert Research Center.

### 2. Phytochemical and elemental analysis of *Chenopodium* species

The crude fiber and ash were measured using the methodology outlined by **Maynard** [40] **and James** [41]. As stated by **Cherry** [42], Nelson’s reagent was used to estimate the total amount of carbohydrates calorimetrically. The total nitrogen content was multiplied by 6.25 to get the crude protein contents, which were determined as total-N using the Keldahle method [43]. The flavonoids were quantified by colorimetric method [44], total phenolics content was determined using Folin Ciocalteu reagent [45,46], the total polyphenol contents were determined according to **Folin and Denis** [46], tannins were determined by spectrophotometer [47], the saponins content was determined based on the method of **Nguyen et al.,** [48], total terpenoids was determined by **Ghorai et al.** [49], and total alkaloids determined by the method of **Shamsa et al.,** [50].

### 3. Aqueous plant tissue extracts

The underground and aboveground parts of the plant were chopped into small pieces, air-dried, and then ground into a fine powder. A total of 100 grams of plant material was extracted in 1000 mL of sterile distilled water using a rotary shaker for 12 hours. The fibers were removed from the mixture using nylon filtration. The resulting mixture was centrifuged at 3000 rpm for 15 minutes, then sterilized using a 0.22 µm pore-size micro-filter. This filtrate represented a 10% (10 g dry weight/100 mL). The filtrate was diluted with distilled water to prepare solutions of 1%, 3%, 5%, 7%, and 9% concentrations, while distilled water alone was used as the control. Ten sterilized seeds were placed in each 9-cm petri dish lined with two layers of filter paper, and 10 mL of the corresponding leachate concentration was added. The petri dishes were covered to minimize evaporation and arranged in a completely randomized design at room temperature (25 ± 2°C) with five replicates per treatment. The experiment was repeated three times. After seven days, seed germination and seedling growth (shoot and root) were recorded. Water-soluble allelochemicals, including coumaric acid (Rt = 31.4, MH + ; 165), ferulic acid (Rt = 35.2, MH + ; 195), quercetin (Rt = 21.2, MH + ; 303), kaempferol (Rt = 28.5, MH + ; 287), and apigenin (Rt = 38, MH + ; 171) were identified based on retention times (Rt) and molecular ion peaks. For chemical analysis, 10 mL of the water extract was freeze-dried, then re-dissolved in 10 mL of absolute methanol. The solution was passed through a 0.22 µm pore micro-filter for final purification before being injected into the LC-ESI-MS (Waters system, USA) for identification, following the method described by El-Sadek et al. [51].

### 4. Decayed plant tissue residues

Chenopodium species were collected, and their vegetative parts were chopped into 2 mm pieces. These pieces were air-dried plant part and mixed into the soil at three application rates: 1%, 3%, and 5% (w/w). Pots were filled with sand soil, either mixed with Chenopodium residues or without residues as a control. Water was added to the pots to achieve field capacity before planting. After 24 hours, wheat (*Triticum aestivum*) and faba bean (*Vicia faba*) seeds were sown into the pots, each containing 10 kg of sieved sand (10 mm sieve) from the Alexandria Desert Road. The soil properties included a pH of 7.8, total soluble salts of 1.13 ds/m, 0.18% organic matter, and 1.12% calcium carbonate (CaCO3). The cation concentrations were 0.24 meq/L for calcium, 0.2 meq/L for magnesium, 0.156 meq/L for sodium, and 0.178 meq/L for potassium, while anions included 0.146 meq/L of bicarbonate and 0.056 meq/L of sulfate, as reported by Sparks et al. [52].

Two days after residue incorporation, 25 seeds were sown in each pot. The pots were placed in a greenhouse with an average temperature of 25 ± 5°C and a 16-hour photoperiod. Irrigation was carried out every three days to maintain soil moisture at field capacity. The pots were arranged in a completely randomized block design, with five replicates for each treatment.

#### 4.1 Germination and growth measurements.

The plants were harvested 28 days after sowing, following irrigation to aid in separating the roots and shoots. Daily germination rates were recorded to calculate germination indices. Root and shoot lengths were measured, and biomass was determined after drying the plant samples in an oven at 80°C for 24 hours. Total chlorophyll content was measured using a SPAD-502 Chlorophyll Meter on fresh plant tissue.

#### 4.2 Determination of phytohormones.

Abscisic acid (ABA), gibberellic acid (GA), and indole-3-acetic acid (IAA) were extracted, purified, and quantified using an HPLC with a photodiode array detector at ʎmax 245 nm (HPLC Ultimate 3000, Thermo Dionex, Germany), following the procedures of Unyayar et al. [53].

#### 4.3 Determination of total proteins and anthocyanins.

Total nitrogen was determined using the Kjeldahl method [43], with the resulting values multiplied by 6.25 to calculate crude protein content. Anthocyanin concentrations were measured at 530 and 690 nm, and calculations followed the method of Mancinelliet al.[54].

#### 4.4 Mineral concentration analysis.

Micronutrient concentrations, including magnesium (Mg), manganese (Mn), zinc (Zn), and iron (Fe), were measured by atomic absorption spectrometry using a UNICAM 929 AA spectrometer. Macronutrient analysis, including potassium (K), calcium (Ca), and sodium (Na), was conducted using flame photometry (Jenway PFP7), following the method described by Cottenie et al. [55].

#### 4.5 Total phenolics in soil.

The total phenolic content of the soil was determined using the Folin-Ciocalteu reagent and measured at 700 nm using a spectrophotometer, following the method of Swain and Hillis [56].

#### 4.6 Soil pH and electrical conductivity.

Soil pH was measured using a soil-to-water ratio of 1:2, following Thomas [57]. Electrical conductivity (dS/m) was determined using the method of Rhoades [58].

#### 4.7 Soil microorganisms.

Total microbial counts in the soil were determined using nutrient agar medium, and CO2 evolution (mg CO2/100g dry soil/24 hours) was used as an indicator of microbial activity, following the method of Jacobs et al. & Atef and Nannipieri [59,60].

### 5. Allelopathy of the essential oils

#### 5.1. Extraction of essential oil.

A Clevenger apparatus was used to hydro-distill 500 g of dried, shaded vegetative parts of weeds for 12 hours to extract the essential oil. The yields of essential oils of *C. ambrosioides*, *C. murale*, and *C. album* were 0.54, 0.56, and 0.48% (v/w). It was stored at 4 °C in a sealed vial for subsequent GC-MS analysis and allelopathic activity assays.

#### 5.2. Bioassays of essential oils.

Essential oils of *C. album*, *C. murale*, *C. ambrosioides* at concentrations of 0 (control), 1, 2.5, 5 and 10 µ L were applied to half-circle filter paper covered with 25 g of sand. The seeds of *C. arvensis* and *B. rigidus* were placed on the other side of the dish with 5 ml of sterile distilled water. These Petri dishes were incubated alternating 25.0 ± 0.2 °C under 16 hr in light and arranged in a completely randomized design with five replications of each treatment, and the experiment was repeated twice. After 7 days, germination and seedling length data were recorded and 50% inhibition was determined.

#### 5.3. Identification of essential oil components.

The identification of volatile components in the oils was performed by gas chromatography coupled with a mass spectrometer detector (Thermo Scientific Corp., USA). GC-MS was performed with a fused silica capillary column with Rtx-5MS stationary phase, (30 m x 0.25 mm 0.25 μm film thickness). Helium was used as the carrier gas. The temperatures were programmed to increase by 3 ºC per minute, reaching a maximum temperature of 240 ºC while, 220 ºC in the injector and 300 ºC in the detector. Compounds were identified by comparing the mass spectra with the equipment database (NIST Spectral Mass Library), and by comparing them with the literature [61,62] and matching authentic samples [63,64].

### 6. Data analysis

The data were subject to analysis by ANOVA using IBM SPSS 21(IBM, Armonk, NY, USA). Means were compared using Duncan multiple range tests to identify a significant level of P ≤ 0.05. Dose-response curves were generated by f = min + (max-min)/(1 + 10^(logEC50-x)) using sigma plot software 12.5.

## Results

### Elemental and phytochemical analysis of *C. album, C. murale* and *C. ambrosioides* species

The phytochemical analysis revealed that *C. album, C. murale,* and *C. ambrosioides* contained ash, crude fiber, total carbohydrates, total tannins, polyphenols, terpenoids, alkaloids, saponins and flavonoids, and the elemental composition of calcium, iron, manganese, zinc, magnesium, sodium, phosphorus, potassium and total nitrogen was determined and is presented in Table 1.

**Table 1:** Elemental and Phytochemical analysis of collected *C.album, C. murale* and *C. ambrosioides* species.

	*C. album*	*C. murale*	*C. ambrosioides*
Crude fiber (%)	2.500 ± 0.95	2.300 ± 0.72	3.107 ± 0.65
Ash (%)	9.440 ± 1.53	11.520 ± 1.53	11.750 ± 0.75
Total carbohydrate (mg/g)	6.513 ± 1.77	5.418 ± 1.00	5.365 ± 0.58
Total tannins (mg/g)	12.476 ± 1.50	12.210 ± 2.022	12.893 ± 1.36
Poly phenols (mg/g)	53.480 ± 2.59	53.769 ± 3.21	56.535 ± 1.80
Terpenoides (mg/g)	1.370 ± 0.50	1.410 ± 0.27	1.472 ± 0.25
Alkaloids (mg/g)	1.230 ± 0.90	1.241 ± 0.23	1.331 ± 0.29
Saponins (mg/g)	1.289 ± 0.33	1.294 ± 0.17	1.386 ± 0.17
Flavonoids (mg/g)	2.634 ± 0.54	1.612 ± 0.18	2.649 ± 0.36
Total nitrogen (%)	0.728 ± 0.41	0.612 ± 0.10	0.623 ± 0.19
Calcium (mg/Kg)	66.025 ± 5.77	57.146 ± 2.58	42.863 ± 3.29
Iron (mg/Kg)	1.144 ± 0.21	1.188 ± 0.17	1.175 ± 0.021
Magnesium (mg/Kg)	72.613 ± 3.67	57.510 ± 5.47	66.822 ± 4.24
Manganese (mg/Kg)	20.292 ± 1.14	29.516 ± 1.50	22.799 ± 1.87
Phosphorus (mg/Kg)	51.906 ± 3.50	41.773 ± 2.76	39.731 ± 3.10
Sodium (mg/Kg)	196.250 ± 4.75	168.746 ± 5.48	175.250 ± 5.64
Potassium (mg/Kg)	423.125 ± 10.43	407.727 ± 7.21	413.750 ± 9.16
Zinc (mg/Kg)	0.474 ± 0.14	0.381 ± 0.08	0.356 ± 0.12
F (p value)	3275(0.00)	3137(0.00)	2979(0.00)

### 2. Allelopathy of *C. album, C. murale* and *C. ambrosioides* via leachates from water extracts

Water extracts of *C. album, C. murale* and *C. ambrosioides* were utilized to compare the leachate potential of their vegetative and subterranean parts against germination and seedling growth of *T. aestivum and V. faba* Table 2. Based on EC_50_ (g/100 ml distilled water), the primary allelopathic effect was observed in vegetative and subterranean parts. *T. aestivum* and root length were identified as the most susceptible crops and parameters to these extracts. For *C. album*, the vegetative and subterranean parts extracts showed EC_50_ values in root length by 3.04, and 3.31(*T. aestivum*), 3.28 and 3.44 (*V. faba)*, respectively.

**Table 2:** Leachates activity and EC_50_ (g. 100 ml^-1^D.W.) of *Chenopodium species* against *T*. *aestivum* and *V. faba* germination and seedling growth.

		*C. album*			*C. murale*			*C. ambrosioides*		
Target species	Extract parts	G%	SL	RL	G%	SL	RL	G%	SL	RL
T. aestivum	Vegetative	5.38 ± 0.42	3.92 ± 0.48	3.04 ± 0.25	4.73 ± 0.37	4.15 ± 0.32	2.79 ± 0.17	4.59 ± 0.22	3.44 ± 0.32	2.64 ± 0.11
	Subterranean	5.55 ± 0.58	4.21± 0.34	3.31 ± 0.18	5.17 ± 0.52	4.46 ± 0.34	3.14 ± 0.28	4.29 ± 0.29	3.55 ± 0.26	2.99 ± 0.17
*V. faba*	Vegetative	5.49 ± 0.47	4.10 ± 0.48	3.28 ± 0.54	4.81 ± 0.48	4.26 ± 0.27	3.87 ± 0.34	4.46 ± 0.19	3.95 ± 0.19	3.25 ± 0.26
	Subterranean	5.90 ± 0.35	4.40 ± 0.37	3.44 ± 0.15	5.19 ± 0.51	4.73 ± 0.33	3.58 ± 0.31	4.93 ± 0.21	3.86 ± 0.53	3.39 ± 0.28
F (p value)										
Species		13.98 **	3.53 **	29.02***	16.34***	10.02**	31.55**	23.29 **	14.20 **	35.56 ***
Extract parts		52.66 ***	19.32 ***	36.61***	43.67***	27.85***	23.45***	20.51 ***	44.13 ***	53.83 ***
Conc.		377.17 ***	449.68 ***	559.49***	153.52***	267.45***	414.85***	123.04 **	323.63 **	4514.67 ***
Species x Extract parts x Conc.		5.94 *	3.16 *	9.11**	6.89**	4.40*	11.67***	5.54 **	4.97 *	14.43**

SL = Shoot length (cm), RL = Root length (cm) and G% germination %, Conc. = concentration, * = P ≤ 0.05 to 0.03; two ** = P ≤ 0.01 to 0.02; three *** = P ≤ 0.001; n s = not significant (P ≥ 0.05).

**Fig 1:**
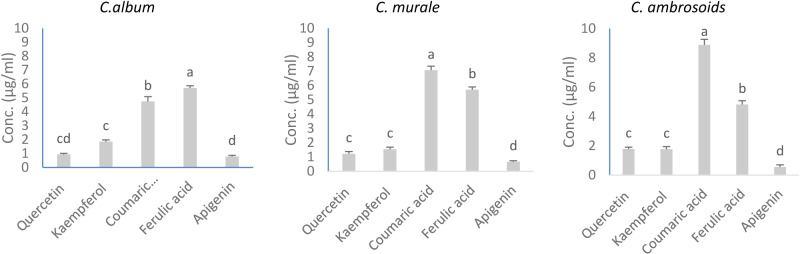
Qualitative and quantitative determination of allelochemicals in water extract of *C. album*, *C. murale* and *C. ambrosioides* using HPLC (DAAD).

For *C. murale* vegetative and subterranean parts extract, it shows EC_50_ values in root length of 2.79, and 3.14(*T. aestivum*), 3.87 and 3.58 (*V. faba)*, respectively. Similarly, *C. ambrosioides* vegetative and subterranean parts extract, it shows EC_50_ values in root length by 2.64, and 2.99 (*T. aestivum*), 3.25 and 3.39(*V. faba)*, respectively. A significant interaction effect of species × plant parts × concentration was recorded from *C. album* aqueous leachates in germination (F = 5.94, *P* ≤ 0.03), shoot length (F = 3.16, *P* ≤ 0.002), and root length (F = 9.11, *P* ≤ 0.00), and from *C. murale* leachates in germination (F = 6.89, *P* ≤ 0.02), shoot length (F = 4.40, *P* ≤ 0.02), and root length (F = 11.67, *P* ≤ 0.000), as well as from *C. ambrosioides* leachates in germination (F = 5.54, *P* ≤ 0.01), shoot length (F = 4.97, *P* ≤ 0.02), and root length (F = 14.43, *P* ≤ 0.000), respectively. The major water-soluble allelochemicals, responsible for most of the allelopathic activity of Chenopodium species, were determined by LC-MS. The water extracts exhibited the highest quantities of allelochemicals, with coumaric acid and ferulic acid showing concentrations of 4.74 and 5.72 μg ml^-1^ in *C. album*, 7.07 and 5.71 μg ml^-1^ in *C. murale*, and 8.88 and 4.82 μg ml^-1^ in *C. ambrosoids*, respectively. In contrast, apigenin was the least abundant compound in all three extracts Fig 1.

### 3. Allelopathy of *C. album, C. murale* and *C. ambrosioides* species decayed residues in soil

The effect of Chenopodium species’ decayed residues in *T. aestivum* and *V. faba* seed germination is shown in Table 3. Generally, incorporation of *C. album*, *C. murale* and *C. ambrosioides* residues in soil substantially enhanced germination at the lower concentration of 1% (w/w) compared with the control treatment (without weed residues). C*. murale and C. album* at 1% achieved the maximum germination by 95.16 and 94.12% respectively. However, Soil incorporation with higher concentrations of 3% and 5% (w/w) resulted in a remarkable reduction in the germination of both crops. *C. ambrosioides* caused the greatest decrease in *V. faba* germination by 50.05%. The time to 50% emergence ranged from 5.62 to 4.91 days in the control and at the highest residue concentration (5%) for *T. aestivum*, and from 6.2 to 5.97 days for *V. faba*. The same trend was observed in the germination rate which decreased by increasing *Chenopodium* species residue incorporation.

**Table 3:** Allelopathic effect of decayed residues of *Chenopodium species* on *T. aestivum* and *V. faba* germination traits.

Extract species		*C. album*		*C. murale*		*C. ambrosioides*	
Conc. (%)		*T. aestivum*	*V. faba*	*T. aestivum*	*V. faba*	*T. aestivum*	*V. faba*
Control	*G max	94.11^a^ ± 0.27	90.99^a^ ± 0.23	92.93^a^ ± 0.36	89.04^ab^ ± 0.32	90.13^a^ ± 0.34	89.99^ab^ ± 0.28
	G rate	1.011 ± 0.03	0.974 ± 0.03	1.093 ± 0.04	1.052 ± 0.04	1.024 ± 0.04	1.013 ± 0.03
	T_50_	5.654 ± 0.03	6.115 ± 0.03	5.723 ± 0.047	6.206 ± 0.042	5.629 ± 0.05	6.133 ± 0.04
1%	G max	94.12^a^ ± 0.41	91.17 ^a^ ± 0.28	95.16^a^ ± 0.32	92.15 ^a^ ± 0.26	92.14^a^ ± 0.34	91.18^a^ ± 0.28
	G rate	1.036 ± 0.05	1.033 ± 0.03	1.144 ± 0.04	1.013 ± 0.03	1.031 ± 0.04	0.943 ± 0.03
	T_50_	5.713 ± 0.05	5.851 ± 0.04	5.4034 ± 0.00	5.812 ± 0.03	5.789 ± 0.04	6.114 ± 0.03
3%	G max	83.05^b^ ± 0.17	74.73^c^ ± 0.31	83.05^b^ ± 0.17	70.74^bc^ ± 0.32	79.04^c^ ± 0.16	68.75^c^ ± 0.33
	G rate	1.150 ± 0.02	1.078 ± 0.04	1.150 ± 0.02	1.129 ± 0.05	1.078 ± 0.02	1.05 ± 0.01
	T_50_	5.479 ± 0.03	6.131 ± 0.05	5.479 ± 0.03	6.107 ± 0.06	5.472 ± 0.02	6.093 ± 0.06
5%	G max	55.07^d^ ± 0.23	52.03^d^ ± 0.27	58.06^d^ ± 0.31	52.03^d^ ± 0.14	53.06^d^ ± 0.13	50.05^d^ ± 0.09
	G rate	0.851 ± 0.02	0.797 ± 0.03	0.807 ± 0.01	0.797 ± 0.07	0.856 ± 0.02	0.821 ± 0.02
	T_50_	4.942 ± 0.10	5.944 ± 0.03	4.977 ± 0.05	5.944 ± 0.09	4.919 ± 0.03	5.972 ± 0.02

Duncan letters (a, b, …) indicate the similarity and difference between concentration, Gmax = The maximum germination percentages, T_50_ = Time to 50% emergence, G rate = germination rate.

Decayed residues of *C. album, C. murale* and *C. ambrosioides* at concentrations of 0, 1, 3, and 5% (w/w) in soil were assessed for their effects on *T. aestivum* and *V. faba* plants and soil parameters over one month Tables 4, 5. Generally, stimulatory effects were observed at the lowest decayed residue concentrations (1% w/w) in most tested plant parameters, while inhibitory effects were noted at higher residue concentrations (3 and 5%).

**Table 4:** Biochemical and elemental components of plant affected by decayed residues of *Chenopodium species.*

	Conc. %(w/w)	Biomass dry weight (g)	Protein (%)	Anthocyanin (%)	total chlorophyll (SPAD)	AB (µg/g)	IAA (%)	GA (%)	N %	p (ppm)	K (ppm)	Mn (ppm)	Zn (ppm)	Fe (ppm)	Mg (ppm)	Ca (ppm)	Na (ppm)
		*C. album*															
*T. aestivum*	0	0.133	7.59	9.87	46.67	101.12	266.12	46.56	0.619	148.93	119.10	0.546	0.593	2.09	63.97	0.061	21.89
	1	0.134	7.84	11.08	45.67	100.61	264.84	47.97	0.623	150.61	127.22	0.572	0.612	2.32	67.92	0.074	21.94
	3	0.122	5.65	6.45	35.00	91.46	231.06	46.68	0.509	132.98	67.81	0.335	0.477	1.57	23.26	0.003	7.08
	5	0.117	3.32	5.14	31.00	40.87	81.61	43.21	0.469	111.36	49.21	0.063	0.147	1.47	11.35	0.003	5.37
*V. faba*	0	0.562	10.60	14.38	38.00	132.77	283.11	72.51	0.73	190.66	119.08	1.118	1.137	2.11	66.43	0.022	23.11
	1	0.568	10.73	17.53	37.00	140.70	286.04	75.35	0.80	204.16	125.35	0.646	1.028	2.10	55.50	0.0148	23.99
	3	0.494	7.28	9.49	28.00	109.52	197.29	46.40	0.72	132.30	62.49	0.511	0.728	1.92	26.38	0.0008	8.197
	5	0.481	4.43	6.36	26.00	37.65	52.58	39.21	0.61	103.70	47.69	0.258	0.602	1.62	14.63	0.0015	7.142
		*C. murale*															
*T. aestivum*	0	0.140	7.71	9.47	46.00	102.65	283.11	52.43	0.609	159.07	119.26	0.570	0.583	2.04	62.84	0.061	19.8
	1	0.145	7.91	11.37	46.33	100.54	286.04	55.27	0.623	160.75	129.94	0.531	0.359	2.06	59.92	0.060	22.7
	3	0.119	5.55	5.25	34.00	99.48	197.29	46.40	0.506	132.20	77.97	0.461	0.267	2.02	22.03	0.005	6.424
	5	0.112	2.96	4.34	31.00	37.65	72.66	42.22	0.466	110.71	47.28	0.399	0.188	0.39	11.37	0.006	5.575
*V. faba*	0	0.572	10.361	14.38	37.00	139.32	287.16	77.49	0.725	192.02	116.08	1.081	1.697	2.35	58.46	0.044	19.36
	1	0.578	10.387	14.82	37.00	142.95	280.50	78.39	0.805	189.33	125.91	1.109	1.217	2.81	56.55	0.041	24.13
	3	0.484	6.808	8.49	31.00	134.46	188.93	52.67	0.710	155.64	72.07	0.982	1.389	2.19	26.12	0.036	10.21
	5	0.471	4.230	6.21	27.00	34.54	41.08	38.75	0.596	122.00	46.33	0.083	0.106	0.68	7.67	0.005	3.09
		*C. ambrosoids*															
*T. aestivum*	0	0.135	6.03	9.36	48.00	101.12	256.08	46.56	0.619	146.90	116.84	0.396	0.364	2.12	62.15	0.062	23.01
	1	0.136	6.21	11.28	48.00	100.61	254.80	47.97	0.627	150.61	127.14	0.255	0.192	1.14	62.48	0.069	23.57
	3	0.111	5.79	4.34	36.00	91.46	231.06	45.67	0.515	130.88	64.35	0.378	0.396	1.07	24.45	0.069	10.12
	5	0.105	2.80	3.23	30.00	30.83	61.53	40.20	0.474	107.92	43.06	0.149	0.078	0.73	11.01	0.004	2.687
*V. faba*	0	0.572	9.59	15.38	34.00	126.10	258.49	70.51	0.724	170.38	119.12	0.796	0.731	2.28	63.08	0.022	21.84
	1	0.669	10.73	15.52	34.00	132.87	261.96	74.18	0.787	191.99	125.64	0.901	0.938	2.59	52.48	0.024	24.81
	3	0.443	6.28	7.49	31.00	104.01	146.87	48.42	0.703	156.25	63.40	0.788	0.825	1.78	27.55	0.025	13.73
	5	0.430	3.44	6.16	28.00	27.29	50.04	34.96	0.590	122.48	35.53	0.532	0.278	1.10	11.54	0.003	1.392

**Table 5:** Biochemical and elemental components of soil affected by decayed residues of *Chenopodium species.*

	Conc. % (w/w)	N %	p (ppm)	K (ppm)	pH	EC (ds/m)	Total phenolic acids (µg/g)
		*C. album*					
*T. aestivum*	0	0.010	10.46	163.65	8.0	0.883	7.741
	1	0.011	12.09	201.38	8.0	0.883	24.721
	3	0.014	13.35	386.74	8.1	1.070	41.730
	5	0.015	16.17	398.81	8.1	1.172	48.887
*V. faba*	0	0.013	11.31	157.83	8.1	0.894	7.74
	1	0.016	14.43	273.40	8.1	0.900	25.04
	3	0.017	17.09	456.10	8.1	1.132	35.10
	5	0.018	18.30	471.93	8.1	1.184	49.14
		*C. murale*					
*T. aestivum*	0	0.010	9.92	153.77	8.0	0.883	7.74
	1	0.011	11.85	197.43	8.0	0.886	24.72
	3	0.013	14.26	381.07	8.1	1.100	34.56
	5	0.015	14.41	383.48	8.1	1.182	50.40
*V. faba*	0	0.013	11.09	148.06	8.1	0.894	7.74
	1	0.016	14.15	268.04	8.1	0.903	25.45
	3	0.016	17.46	466.10	8.1	1.134	35.90
	5	0.018	17.21	471.93	8.1	1.188	51.21
		*C. ambrosoids*					
*T. aestivum*	0	0.011	11.05	187.95	8.0	0.883	7.74
	1	0.014	11.73	195.46	8.0	0.883	24.72
	3	0.015	13.50	384.41	8.1	1.081	42.15
	5	0.016	16.50	407.15	8.2	1.179	54.72
*V. faba*	0	0.013	10.98	153.08	8.1	0.907	7.74
	1	0.015	14.01	265.36	8.1	0.910	25.48
	3	0.016	15.27	456.10	8.2	1.143	44.59
	5	0.017	17.21	471.93	8.2	1.196	54.62

Conversely, soil parameters showed inverse effects, with increases in the measured parameters corresponding to higher concentrations of Chenopodium species residues. The plant parameters, including dry weight, protein%, anthocyanin%, total chlorophyll, AB, IAA, GA, N%, P, K, Mn, Zn, Fe, Mg, Ca, and Na, correlated negatively with increasing concentrations of decayed residues, ranging from -0.695 to -0.959 for *Triticum aestivum* and -0.561 to -0.896 for *Vicia faba*, respectively. However, the soil parameters of N%, P, K, pH, EC and soil total phenolic acids correlated positively with decayed residues in *T. aestivum*, (0.878 to 0.975) and *V. faba* (0.667 to 0.966) respectively. The strongest negative correlation of soil EC with plant N% was -0.996 for *T. aestivum* and -0.987 for plant K in *V. faba*. The strongest negative correlation of soil phenolic compounds with chlorophyll contents was -0.915 for *T. aestivum* and -0.902 for *V. faba*. Additionally, the plant macro elements N, P, and K correlated negatively with soil N, P, K ranging from -0.855 to -0.967 for *T. aestivum* and -0.559 to - 0.900 for *V. faba*.

The statistical analysis of *C. album*, *C. murale* and *C. ambrosioides* decayed residue effects on *T. aestivum* and *V. faba* germination, seedling growth and biochemical composition included protein %, pigment (anthocyanin and chlorophyll) and phytohormones contents including abscisic acid (AB (, indole acetic acid (IAA (and gibberellic acid (GA) is shown in Table 6. The analysis demonstrated a higher effect of residue concentration than residue species in both *T. aestivum* and *V. faba* attributed parameters. The responses of plant traits including germination, shoot length, root length, protein, anthocyanin and chlorophyll contents to the effect of residue species were higher in *T. aestivum* crop than *V. faba* traits and vice versa in AB, IAA and GA contents to the tested species and concentrations of incorporated residue. The interaction effect of plant species × extract type ×concentration was significantly in total biomass dry weight (F = 40.64, *p *≤ 0.00), shoot length (F = 63.95, *p *≤ 0.00), root length (F = 75.70, *p *≤ 0.00), germination (F = 3.73, *p *≤ 0.03), protein% (F = 79.19, *p *≤ 0.00), anthocyanin (F = 61.82, *p *≤ 0.00), chlorophyll (F = 4.27, *p *≤ 0.04), AB (F = 29.72, *p *≤ 0.00), IAA (F = 57.44, *p *≤ 0.00) and GA (F = 11.04, *p *≤ 0.00) respectively.

**Table 6:** Variance analysis of *Chenopodium species* decayed residues on *T. aestivum* and *V. faba* and biochemical components.

	Total biomass Dry weight	Shoot length	Root length	Germination	Protein%	Anthocyanin	Chlorophyll	AB	IAA	GA
Plant species	1989.39***	3335.82***	2304.22***	10.90**	5659.37***	8758.82***	482.41***	395.20***	260.52***	389.52***
Extract type	12.32**	3169.77***	885.08***	20.59**	3062.56***	2053.35***	12.21 **	1958.34***	1597.71***	1506.61***
Concentration	2182.69***	1496.36***	1186.60***	607.90***	7974.18***	1741.02***	355.74***	1178.72***	1341.33***	2723.86***
Plant species x Extract type	10.50**	51.65***	437.07***	15.01**	157.62***	273.60***	4.25*	655.97***	282.97***	112.60***
Plant species x Concentration	1012.18***	1572.27***	2191.06***	5.64*	479.20***	3274.77***	29.02**	5171.24***	2046.13***	1272.69***
Extract type x Concentration	176.23***	414.48***	159.56***	1.69^ns^	236.85***	550.00***	1.51 ^ns^	437.38***	335.88***	183.34***
Plant species x Extract type x Concentration	40.64***	63.95***	75.70***	3.73*	79.19***	61.82***	4.27*	29.72***	57.44***	11.04***

The variance analysis of *C. album*, *C. murale* and *C. ambrosioides* decayed residues on the elemental composition of *T. aestivum* and *V. faba* crops is shown in Table 7. The interaction effects of plant species × concentration were significant in nitrogen (F = 8.22, *p *≤ 0.01), phosphorus (F = 23.35, *p *≤ 0.001), and potassium (F = 3.72, *p *≤ 0.03), respectively. As for plant microelements composition, there were interaction effect of plant species × extract type × concentration in plant elements including Ca (F = 131.30, *p *≤ 0.00), Mn (F = 198.84, *p *≤ 0.00), Zn (F = 23.13, *p *≤ 0.00), Fe (F = 16.51, *p *≤ 0.00), and Na (F = 6.97, *p *≤ 0.02) respectively.

**Table 7:** Variance analysis of Chenopodium species decayed residues on *T. aestivum* and *V. faba* elemental composition.

	N	P	K	Ca	Mn	Zn	Fe	Mg	Na
Plant species	456.04***	527.36***	26.45***	64712.13***	58988.92***	2110.72***	25642.32***	28.37***	12.79**
Extract type	0.72^ns^	12.32**	2.89*	9611.53***	4699.23***	474.80***	3694.16***	22.06***	12.72**
Concentration	114.17***	434.74***	205.48***	6873.22***	4923.16***	4553.95***	9039.51***	2204.66***	282.07***
Plant species x Extract type	0.54 ^ns^	3.87*	0.14 ^ns^	1757.44***	2456.18***	447.22***	6163.32***	0.58 ^ns^	3.95*
Plant species x Concentration	8.22***	23.36***	3.72*	599.37***	270.42***	174.03***	758.25***	3.78*	20.27***
Extract type x Concentration	0.09 ^ns^	3.33*	1.17 ^ns^	156.08***	161.51***	214.71***	322.72***	27.65***	43.69***
Plant species x Extract type x Concentration	0.02 ^ns^	2.47 ^ns^	0.05 ^ns^	131.30***	198.84***	23.13***	16.51***	2.49 ^ns^	6.97**

The effects of Chenopodium species incorporated residues, and concentration on soil parameters including pH, EC, total phenolic acid, and macro-elements are shown in Table 8. The role of decayed residues was significant in soil pH from plant species (F = 4.05, *p *≤ 0.04), extract type (F = 6.51, *p *≤ 0.02), and concentration (F = 16.70, *p *≤ 0.00) respectively. At the same time, There was significant effect in soil EC from plant species (F = 5.49, *p *≤ 0.03), and concentration (F = 9.02, *p *≤ 0.01) respectively. These incorporated residues had significant interaction affect in the soil phenolic acids by plant species × concentration (F = 6.89, *p *≤ 0.02). As for the effect of incorporated residue in soil macro-elements, there were significant interactions effect in soil nitrogen from plant species × extract type (F = 9.77, *p *≤ 0.01), plant species × concentration (F = 15.19, *p *≤ 0.00) and extract type × concentration (F = 7.56, *p *≤ 0.02) respectively. While, the significant interaction effect in soil phosphorus displayed by plant species × extract type (F = 6.21, *p *≤ 0.03), plant species × concentration (F = 32.72, *p *≤ 0.00), and extract type × concentration (F = 3.00, *p *≤ 0.04) respectively. Also, There were significant interaction in soil potassium from plant species × extract type (F = 18.24, *p *≤ 0.00), plant species× concentration (F = 3.66, *p *≤ 0.04), and extract type × concentration (F = 10.65, *p *≤ 0.02) respectively. As for the effect of incorporated residues in soil microbes, there were significant effect between plant species× extract type × concentration on total microbes counts (F = 15.64, *p *≤ 0.00) and CO_2_ (F = 7.19, *p *≤ 0.02) respectively.

**Table 8:** Variance analysis of *C. album*,*C. murale* and *C. ambrosioides* decayed residues on soil composition.

	pH	EC	Soil phenols	N	P	K	Microbes count	CO_2_
Plant species	4.05*	5.49*	44.85***	249.75***	705.13***	11.89**	817.17**	214.00***
Extract type	6.51**	0.56 ^ns^	0.31 ^ns^	3.88**	4.04*	117.11***	115.04**	59.84***
Concentration	16.70***	9.02***	30.10***	431.22***	455.62***	324.35***	360.42**	844.24***
Plant species x Extract type	0.13 ^ns^	0.37 ^ns^	2.10 ^ns^	9.77**	6.21*	18.24***	14.25**	25.40***
Plant species x Concentration	1.04 ^ns^	2.12 ^ns^	6.89**	15.19***	32.72***	3.66*	12.12**	13.13**
Extract type x Concentration	1.83 ^ns^	0.09 ^ns^	1.45 ^ns^	7.56**	3.00*	10.65**	2.41 ^ns^	25.74**
Plant species x Extract type x Concentration	0.11 ^ns^	0.07 ^ns^	4.41*	1.55 ^ns^	1.61 ^ns^	5.98*	15.64**	7.19**

#### Allelopathy of *C. album, C. murale* and *C. ambrosioides* species essential oils.

Table 9 indicated the greatest inhibitory effects of essential oils on the shoot length of *C. arvensis* and *B. rigidus* compared to root length and seed germination based on their EC_50_. For *C. album* essential oils, the EC_50_ values (µl per petri dish) in shoot length were 3.47 (*C. arvensis*) and 3.38 (*B. rigidus*). For *C. murale*, the EC_50_ for essential oils in shoot length was 3.38 (*C. arvensis*) and 3.00 (*B. rigidus*), respectively. Meanwhile, the essential oils of *C. ambrosioides* exhibited EC_50_ values in shoot lengths of 3.14 (*C. arvensis*) and 2.98 (*B. rigidus*), respectively. Variance analysis revealed that the most significant interaction inhibitory effect between plant species× concentration in shoot length (F = 14.51, P ≤ 0.000), root length (F = 18.82, P ≤ 0.000), and germination (F = 7.93, P ≤ 0.000) in *C. arvensis.* Also, there was a significant interaction effect in shoot length (F = 18.44, P ≤ 0.000), root length (F = 24.31, P ≤ 0.000), and germination (F = 21.98, P ≤ 0.000) respectively in *B. rigidus*.

**Table 9:** Allelopathic effects of *C.album, C. murale* and *C. ambrosioides* volatile oils on *C. arvensis* and *B. rigidus weeds.*

EC_50_ (µl. petri dish)	*C. arvensis*			*B. rigidus*		
	Shoot length	Root length	Germination	Shoot length	Root length	Germination
*C. album*	3.478 ± 0.28	3.76 ± 0.26	4.023 ± 0.38	3.38 ± 0.13	3.742 ± 0.135	3.81 ± 0.265
*C. murale*	3.38 ± 0.48	3.77 ± 0.60	4.023 ± 0.38	3.00 ± 0.16	3.244 ± 0.26	3.45 ± 0.241
*C. ambrosioides*	3.143 ± 0.21	3.47 ± 0.25	3.763 ± 0.31	2.98 ± 0.11	3.042 ± 0.125	3.22 ± 0.2165
F value			.			
Plant species	35.95 ***	67.96 ***	14.82 ***	15.03 ***	78.49 ***	49.69 ***
Concentration	236.94 ***	312.99 ***	295.47 ***	257.21 ***	274.85 ***	220.10 ***
Plant species x Concentration	14.51 ***	18.82***	7.93 ***	18.44 ***	24.31 ***	21.98 ***

#### The composition of *Chenopodium species* essential oils by GC/MS.

GC/MS analyses of *C. album, C. murale,* and *C. ambrosioides* essential volatile oils were identified in Table 10. The dominant compounds in *C. ambrosioides* oils were ascaridole (8.87%), α-pinene (6.70%), camphor (5.76%), and carvacrol (5.64%), respectively. In *C. murale* oils, the major constituents were carvacrol (10.64%), α-pinene (8.38%), and terpinen-4-ol (7.09%) followed by trans-sabinene (5.39%) and camphene (5.11%), respectively. Meanwhile, C. *album* oils were characterized by carvacrol (8.43%), and germacrene (5.08%), respectively.

**Table 10:** Essential oils composition of *Chenopodium species* analyzed by GC/MS.

No	Rt	Essential oils	*C. album*	*C. murle*	*C. ambrosodies*
1	4.58	β-Myrcene	0.00	0.00	2.66
2	4.79	α-thujene	0.00	0.00	2.98
3	5.24	α-pinene	4.18	4.84	6.70
4	5.81	β-Phellandrene	3.83	3.22	0.00
5	6.07	octanol	5.92	8.38	0.99
6	6.23	β-pinene	3.52	4.84	5.34
7	6.55	Camphor	8.20	5.48	5.76
8	6.82	p-cymene	4.97	3.55	5.46
9	7.14	6-Methyl-5-hepten-2-one	4.56	5.48	4.42
10	7.45	Carvacrol	8.43	10.64	5.64
11	7.63	1,8-cineole	5.76	3.22	1.81
12	8.56	Cis-ocimene	0.00	0.00	6.13
13	9.06	γ-terpinene	0.00	0.00	4.81
14	10.14	Linalool	6.15	7.42	1.95
15	10.45	Pinane-2-ol	0.00	0.00	0.83
16	11.16	β-Caryophyllene	0.00	0.00	1.01
17	11.64	Citronellal	0.35	1.00	0.22
18	11.85	Borneol	0.00	0.00	1.18
19	12.05	Terpinen-4-ol	7.89	7.09	3.53
20	12.46	α-terpineol	3.52	0.00	3.75
21	12.65	Citronellol	2.47	0.97	0.39
22	12.78	Ascaridole	1.93	1.93	8.87
23	13.05	trans 4 carene	0.73	4.19	3.21
24	13.45	Linalyl acetate	0.00	0.00	0.83
25	13.97	Geranial	4.31	2.90	2.68
26	14.65	Germacrene D	0.00	0.00	1.74
27	15.20	Thymol	6.53	4.84	5.25
28	15.64	Ethyl cinnamate	2.25	5.48	0.00
29	15.78	Acetyl eugenol	0.22	4.84	0.00
30	16.32	Phytol	4.85	3.55	3.41
31	16.74	trans-Ascaridol glycol	0.00	0.00	3.21
32	16.87	-p-Mentha-2,8-dien-1-ol	0.00	0.00	0.59
33	17.32	2,3-Epoxy carvone	2.63	2.90	0.00
34	18.2	1,2,3,4-Tetrahydroxy-p-menthane	2.66	3.22	1.85
35	18.9	Benzyl benzoate	4.15	0.00	2.78
36	19.84	Heptacosane	0.41	1.16	0.59
Total			100	100	100

## Discussion

The study aimed to assess the allelopathic effects of *Chenopodium album*, *C. murale*, and *C. ambrosioides*, which are widely distributed and known to impact various ecosystems. These species, part of the Chenopodiaceae family, are prevalent across different habitats in Egypt (64). In Egypt, the Chenopodium genus is represented by 9 species that inhabit variable habitats of different regions as a weed of cultivation and waste ground in the Mediterranean Coast and Nile Delta of Egypt [39,65,66]. To investigate their biological impacts, this study examined their allelopathic potential through leachates, decayed residues, and volatilization, focusing on both monocotyledonous and dicotyledonous crops. Allelopathy is a process still little studied globally, considering the diversity of biochemical compounds through which plants can interact with each other [67,68]. Therefore, it is essential to identify the concentration at which each species responds to allelochemicals and understand the interaction for use in weed management [69].

Analysis of leachate via water extracts revealed a potent allelopathic effect of Chenopodium species on the tested crop attributes, significantly influenced by plant parts, concentrations, and their interactions. Notably, aboveground parts exhibited higher inhibitory effects compared to subterranean parts. They demonstrated stimulatory effects at the lowest concentrations (1%) and inhibitory effects at higher concentrations (3%, 5%, 7%, and 9%) on the germination and seedling growth of *Triticum aestivum* and *Vicia faba* crops. The strongest allelopathic potential was observed in *C. ambrosioides* in both *T. aestivum* and *V. faba traits*, followed by *C. murale*. The findings clearly indicate that the allelopathic effects observed in Chenopodium species are heavily influenced by their chemical composition. The water extracts of *C. album*, *C. murale*, and *C. ambrosioides* contained significant quantities of phenolic compounds, such as hydroxycinnamic acids (coumaric and ferulic acids), flavonoids (quercetin, kaempferol, and apigenin), terpenoids, alkaloids, and saponins. These allelochemicals inhibit the growth of other plants through various biochemical pathways [70,71]. The extracts of *C. album*, *C. murale*, and *C. ambrosioides* showed profound potential in suppressing the in vitro growth of *Macrophomina phaseolina*, a soil-borne fungal plant pathogen [72]. *C. album and C. murale* exhibit allelopathic properties that suppress the germination and growth of native vegetation and crop plants [12]. Allelochemicals soluble in Chenopodium species proved strong inhibitors to several species tested: *Trifolium alexandrinum*, *Triticum aestivum*, *Melilotus indicus*, *Lycopersicon esculentum*, and *Cucumis sativus* [31,73]. Chenopodium species are rich in secondary metabolites with known allelopathic properties. The presence of phenolic compounds like tannins, flavonoids, and polyphenols, identified as key allelochemicals, further supports the hypothesis that these species possess the ability to inhibit the growth of other plants [71,74]. These compounds play a critical role in altering soil microbial communities and plant competition dynamics, giving Chenopodium species a competitive edge over other plant species, including crops and weeds [72,75]. Further studies have demonstrated that phenolic allelochemicals can disrupt plant-plant interactions and alter the microbial composition of soils, contributing to plant invasion and competition [76,77]. This ecological advantage is critical for Chenopodium species, as it enhances their ability to outcompete both weeds and crops [70,74,78]

Chenopodium species (*C. album*, *C. murale*, and *C. ambrosioides*) caused significant inhibition in the germination of *Avena fatua* [69,71]. Germination is less sensitive to allelopathic effects, despite its widespread use in allelopathy bioassays [79]. *C. album* and *C. murale* are allelopathic and, thus, suppress the germination and growth of native vegetation and/or crop plants [70]. The allelopathic effects of *C. album* on wheat (*T. aestivum*) included reduced germination (%), and decreased shoot and root length [71]. The aqueous extract of *C. album* inhibited the germination and growth of wheat [75]. *C. murale* aqueous extract affected seed germination of *Triticum aestivum*, *Zea mays*, *Cicer arietinum*, and *Vigna radiata* exhibited pronounced allelopathic inhibitory effects proportional to the concentration of extracts [74]. Plant leachates contribute inhibitors (e.g., phenolic acids) and promoters (e.g., nitrates) into the soil environment [77,80,81].

The presence of these compounds in higher concentrations, as confirmed by LC-MS analysis, suggests that allelopathy in Chenopodium species is primarily mediated by the release of phenolic compounds. The inhibitory effects were more pronounced in the aboveground parts of these species compared to the subterranean parts, indicating that the concentration and type of allelochemicals present vary depending on the plant part. This finding aligns with research by [82], which suggests that phenolic-rich plants tend to exert stronger allelopathic effects through their leaves than through their roots. The physiological responses of *T. aestivum* and *V. faba* to Chenopodium residues support the notion that allelopathic effects are dose-dependent. At low concentrations (1%), Chenopodium residues stimulated crop growth, promoting seedling elongation, dry matter accumulation, and chlorophyll content. This stimulatory effect is consistent with findings from [83], which showed that low doses of allelochemicals could enhance plant growth. However, at higher concentrations (3% and 5%), the inhibitory effects became more pronounced. The significant reduction in germination rate, shoot, root length, and overall biomass highlights the toxic impact of higher allelochemical concentrations on plant physiological processes. The observed reduction in chlorophyll content can be attributed to disruptions in photosynthetic machinery, a known consequence of phenolic acid exposure [84]. Comparing our findings with existing literature, the allelopathic effects observed in our study align with previous research on the impact of phenolic acids and other allelochemicals on soil properties and plant growth [81,85–87]. Our study extends these findings by demonstrating how different Chenopodium species and their residues affect soil microbial activity and CO_2_ production, offering new insights into their ecological impact and potential applications in weed management. The greatest allelopathic potentials of *C. album*, *C. murale*, and *C. ambrosioides* essential oils were observed in *C. arvensis* and *B. rigidus*, subsequently hindering their seed germination and growth. The highest inhibitory effect was observed with *C. ambrosioides*, followed by *C. murale*. However, *C. album* recorded the lowest effects. Consequently, these essential oils could serve as a herbicidal treatment for suppressing weed seeds. The activity of the essential oil of *C. ambrosioides* is probably related to the large amount of ascaridole, since the other major compound, p-cymene, is recognized as a potent anti-inflammatory with low cytotoxic activity [88]. The volatile oil from *C. ambrosioides* via volatilization exhibited stronger allelopathy on the growth of surrounding plants than via eluviation [81]. The main components of volatile oil from *Chenopodium ambrosioides* L. are p-cymene (29.73%), α-terpinene (9.02%), ascaridole (23.51%), and α-terpinolene or δ-4-carene (31.17%) [88]. Chenopodium residues affect soil parameters, particularly soil pH, EC, and phenolic acid content. Incorporating decayed residues altered soil chemistry, with a negative correlation observed between phenolic acid content and nutrient availability. The increase in soil phenolic acids was particularly detrimental to chlorophyll synthesis in *T. aestivum* and *V. faba*, with strong negative correlations recorded between soil phenolic compounds and chlorophyll content (-0.915 for *T. aestivum* and -0.902 for *V. faba*). This relationship underscores the significant role that allelochemicals play in altering nutrient dynamics in the soil, ultimately affecting plant physiological traits.

Moreover, the interaction between decayed residues and soil microbes was noteworthy. The study found that microbial activity, measured by CO_2_ production and total microbial counts, increased at lower residue concentrations but decreased at higher concentrations. This indicates that while allelopathic compounds can provide a carbon source for microbes at low levels, excessive concentrations may exert toxic effects, reducing microbial diversity and activity [89].

The essential oils of *C. album*, *C. murale*, and *C. ambrosioides* showed promise as potential herbicides, particularly against the problematic weeds *Convolvulus arvensis* and *Bromus rigidus*. The herbicidal potential of *C. ambrosioides* oil, attributed to its high ascaridole content, was especially significant, aligning with previous findings on the herbicidal properties of Chenopodium species [88]. This highlights the potential use of Chenopodium essential oils in developing eco-friendly herbicidal formulations. The allelopathic potential of Chenopodium species, especially through their essential oils and decayed residues, holds significant promise for weed management and sustainable agriculture. However, the effects on soil microbial communities and nutrient dynamics must be carefully managed to avoid negative ecological impacts. Further research should explore the long-term implications of incorporating Chenopodium residues into agricultural systems, particularly with soil health and crop productivity.

## Conclusion

This study evaluated the allelopathic effects of *Chenopodium* species, revealing that *C. ambrosioides* exhibited the strongest allelopathic potential, followed by *C. murale*, while *C. album* showed comparatively fewer effects through leachates and decayed residues against crop species and its essential oil against tested weeds. Our findings indicate that the degree of inhibition observed in crop growth and soil characteristics is significantly influenced by the allelopathic pathways, concentrations, and specific plant parts used. Specifically, leachates from water extracts of Chenopodium species were found to contain higher quantities of hydroxycinnamic acids compared to polyphenolic acids. These polyphenolic acids, including coumaric acid and ferulic acid, have been shown to contribute significantly to the allelopathic effects observed in this study. High concentrations of incorporated residues from these species resulted in markedly negative effects on soil microbial counts, CO2 production, and increased soil phenolic levels, which in turn adversely impacted crop growth and altered elemental and biochemical contents. Conversely, essential oils from Chenopodium species demonstrated considerable biological activity against persistent weeds such as *C. arvensis* and *B. rigidus*, highlighting their potential application as natural herbicides. These results underscore the need for careful management of Chenopodium species to mitigate their adverse effects on cultivated crops and soil health. Implementing strategies to control their allelopathic capabilities can help prevent negative impacts on crop production and soil quality.

## Supporting information

S1 Data Supplementary data 1. (XLSX)
